# Interferon regulatory factor family influences tumor immunity and prognosis of patients with colorectal cancer

**DOI:** 10.1186/s12967-021-03054-3

**Published:** 2021-09-06

**Authors:** Yan-Jie Chen, Shu-Neng Luo, Ling Dong, Tao-Tao Liu, Xi-Zhong Shen, Ning-Ping Zhang, Li Liang

**Affiliations:** 1 Department of Gastroenterology, Zhongshan Hospital, Fudan University, 180 Fenglin Road, Shanghai, 200032 China; 2 Department of Medical Oncology, and Cancer Center, Zhongshan Hospital, Fudan University, 180 Fenglin Road, Shanghai, 200032 China

**Keywords:** Interferon regulatory factor family, Colorectal cancer, Prognosis, Immunotherapy, Tumor immune infiltration

## Abstract

**Background:**

Since interferon regulatory factor (IRF) family functions in immune response to viral infection, its role in colorectal cancer (CRC) has not been inspected before. This study tries to investigate members of IRF family using bioinformatics approaches in aspect of differential expressions, biological function, tumor immune infiltration and clinical prognostic value for patients with CRC.

**Methods:**

Transcriptome profiles data, somatic mutations and clinical information of CRC were obtained from COAD/READ dataset of The Cancer Genome Atlas (TCGA) as a training set. Gene expression data (GSE17536 and GSE39582) were downloaded from the Gene Expression Omnibus as a validating set. A random forest algorithm was used to score the risk for every case. Analyzing gene and function enrichment, constructing protein–protein interaction and noncoding RNA network, identifying hub-gene, characterizing tumor immune infiltration, evaluating differences in tumor mutational burden (TMB) and sensitivity to chemotherapeutics or immunotherapy were performed by a series of online tools and R packages. Immunohistochemical (IHC) examinations were carried out validation in tissue samples.

**Results:**

Principal-component analysis (PCA) suggested that the transcript expression levels of nine members of IRF family differed between normal colorectum and CRC. The risk score constructed by IRF family not only acted as an independent factor for predicting survival in CRC patients with different biological processes, signaling pathways and TMB, but also indicated different immunotherapy response with diverse immune and stromal cells infiltration. IRF3 and IRF7 were upregulated in CRC and suggested a shorter survival time in patients with CRC. Differentially expressed members of IRF family exhibited varying degrees of immune cell infiltration. IHC analysis showed a positive association between IRF3 and IRF7 expression and tumor-infiltrating immune cells, including CD4^+^ T cell and CD68^+^ macrophages.

**Conclusions:**

On account of differential expression, IRF family members can help to predict both response to immunotherapy and clinical prognosis of patients with CRC. Our bioinformatic investigation not only gives a preliminary picture of the genetic features as well as tumor microenvironment, but it may provide a clue for further experimental exploration and verification on IRF family members in CRC.

**Supplementary Information:**

The online version contains supplementary material available at 10.1186/s12967-021-03054-3.

## Background

Colorectal cancer (CRC) is one of the leading causes of cancer-related deaths worldwide [1]. Approximately 1.8 million new CRC cases and > 860,000 CRC-related deaths occurred globally in 2018, making CRC the third most frequent cancer worldwide [1, 2]. CRC develops through a multistep process characterized by accumulated genetic and epigenetic abnormalities that cause genomic instability and mutations in tumor-suppressor and oncogenic genes [3]. Most CRC lesions show little sensitivity to immune-checkpoint inhibitor-based therapies, although immunologic parameters may have prognostic value [4]. Therefore, further research on the tumor immunity of CRC will provide a theoretical basis for developing CRC immunotherapeutic.

Interferons were first discovered as antiviral proteins, and subsequently, interferon regulatory factors (IRFs) were discovered and studied intensively. IRFs are transcription factors participating in interferon gene regulation [5]. The amino termini of IRFs contain a DNA-binding domain (DBD) composed of 115 amino acids (like DBD of Myb) and can bind promoter regions in DNA. The carboxyl termini of IRFs have a variable region that serves various biological functions [6]. Ten IRFs (IRF1 to IRF9 and virus IRF) have been discovered. IRFs are found in various tissues and play important roles in cell-cycle regulation, cell differentiation, apoptosis, and tumor immune regulation [6]. Future studies on IRFs will provide a theoretical basis for their mechanistic roles in tumor development and tumor immunity and for choosing drug therapies.

The roles of IRFs in CRC have not been investigated using bioinformatics analysis. Here, we used public databases to analyze IRF expression levels and mutations in CRC patients to determine distinct prognostic values, study tumor immunity regulation, and identify potential functions of IRFs in CRC. We verified these results via immunohistochemistry (IHC) analysis with our own cohort of CRC patients.

## Methods

### Data acquisition

Data regarding fragments per kilobase million (FPKM) values and microRNA (miRNA)-expression levels of patients with CRC were downloaded from the COAD/READ datasets of The Cancer Genome Atlas (TCGA) Genomic Data Commons website (https://portal.gdc.cancer.gov/) and used as the training dataset. FPKM values were converted to transcripts per million values and divided into mRNA- and long noncoding RNA (lncRNA)-expression groups. “Masked Somatic Mutation” data of patients with CRC were downloaded, pre-processed using VarScan software, and visualized using the R software package, maftools [7]. The clinicopathological features and prognoses of patients with CRC, such as gender, age, and stage, were downloaded from the UCSC Xena website (http://xena.ucsc.edu/). After removing samples with missing clinical information, 597 tumor samples and 51 normal tissue samples were obtained. Table 1 and Additional file 5: Table S6 shows the baseline clinical information of patients with CRC from TCGA-COAD/READ datasets. The likelihood of each response to immunotherapy was predicted using the Tracking of Indels by DEcomposition (TIDE) algorithm (http://tide.dfci.harvard.edu) [8]. Gene expression data from different organizations and in different cell lines were downloaded from TCGA and the Cell Line Cancer Encyclopedia (CCLE) databases (https://portals.broadinstitute.org/ccle/about) to compare IRF expression levels between tumor and normal tissues.

**Table 1:** The baseline information of patients with colorectal cancer (CRC) and scoring interferon regulatory factor (IRF) family by random forest algorithm from The Cancer Genome Atlas (TCGA) database of COAD/READ datasets

Patients from COAD/READ	All patients (n = 597)	Low (n = 298)	High (n = 299)	*P* value
Gender				0.904
Female	277 (46.4%)	139 (46.6%)	138 (46.2%)	
Male	320 (53.6%)	159 (53.4%)	161 (53.8%)	
Age				0.378
< 60	170 (28.5%)	80 (26.8%)	90 (30.1%)	
≥ 60	427 (71.5%)	218 (73.2%)	209 (69.9%)	
Pathologic stage				< 0.001
I	108 (18.1%)	69 (23.1%)	39 (13.1%)	
II	225 (37.7%)	120 (40.3%)	105 (35.1%)	
III	177 (29.6%)	86 (28.9%)	91 (30.4%)	
IV	87 (14.6%)	23 (7.7%)	64 (21.4%)	
T				0.002
T1	19 (3.2%)	11 (3.7%)	8 (2.7%)	
T2	105 (17.6%)	65 (21.8%)	40 (13.4%)	
T3	408 (68.3%)	201 (67.5%)	207 (69.2%)	
T4	65 (10.9%)	21 (7.0%)	44 (14.7%)	
N				< 0.001
N0	342 (57.3%)	191 (64.1%)	151 (50.6%)	
N1	145 (24.3%)	71 (23.8%)	74 (24.7%)	
N2	110 (18.4%)	36 (12.1%)	74 (24.7%)	
M				< 0.001
M0	453 (75.9%)	249 (83.6%)	204 (68.2%)	
M1	85 (14.2%)	22 (7.4%)	63 (21.1%)	
MX	59 (9.9%)	27 (9.0%)	32 (10.7%)	

Gene expression data in GSE17536 [9] and GSE39582 [10] and clinicopathological patient characteristics were downloaded as validation datasets from the Gene Expression Omnibus (GEO) database. The data were downloaded from Homo Sapiens; this platform is based on the GPL570 [HG-U133_PLus_2] Affymetrix Human Genome U133 Plus 2.0 Array. GSE17536 included 177 colon cancer tissue samples, and GSE39582 included 566 colon cancer tissue samples and 19 colon non-tumor tissue samples.

### Genetic characteristics of the IRF family and validation by constructing clinical prediction models

We incorporated the expression levels of IRF family genes into a random forest model. The random forest package of R [11] was used to develop an IRF-based risk-assessment model for patients with CRC. Patients were divided into high- and low-IRF risk groups, based on the median value.

To assess patient prognosis by combining the IRF risk score with clinicopathological features, univariate and multivariate Cox proportional-hazards analyses were used to analyze the independent predictive power of risk scores for overall survival (OS) and disease-free survival (DFS). Subsequently, a survival-prediction nomogram was constructed for patients in TCGA dataset and was validated for patients in the GEO dataset. To quantify differentiation performance, Harrell’s consistency index (C-index) was measured. A calibration curve was generated to evaluate the performance of the line map by comparing the predicted value of the line map with the observed OS rate. In the calibration curve, the abscissa shows the survival rate predicted by the model, and the ordinate shows the survival rate observed. Theoretically, the prediction should be consistent with the observation, which is the diagonal line. However, there is still a gap between the actual process and the theory. The closer the line and the dashed line between the points, the better the consistency of the model. We used the above methods to evaluate the quality of the model.

### Differentially expressed genes (DEGs) and clinical correlation analysis

Data of patients with CRC were downloaded from TCGA and the GEO databases, and the patients were divided into high- and low expression groups, according to the IRF score. The DESeq2 package of R [12] was used to analyze DEGs in both groups, where a log fold-change (logFC) more than 1.0 and *P* value less than 0.05 was considered a threshold value with statistical difference for DEGs.

We compared the expression levels of IRF family genes at different TNM stages. The Human Protein Atlas (HPA, https://www.proteinatlas.org) provides immunohistochemical expression data for nearly 20 different cancers [13] and enables the identification of tumor type-specific differential protein expression patterns, where protein expression levels of all IRF family genes were compared between normal and CRC tissues.

### Functional enrichment analysis and gene set enrichment analysis (GSEA)

Gene Ontology (GO) analysis is commonly used for large-scale functional enrichment research of biological processes (BPs), molecular functions, and cellular components. The Kyoto Encyclopedia of Genes and Genomes (KEGG) is a widely used database containing information regarding genomes, biological pathways, diseases, and drugs. GO and KEGG pathway-enrichment analyses were performed with signature genes using the clusterProfiler R package [14]. A false-discovery rate of < 0.05 was considered statistically significant.

To investigate differences in BPs among different subgroups, GSEA was performed using the gene expression profiles of patients with CRC. GSEA can be used to identify the statistical differences between two groups in a gene set and estimate changes in pathways and BP activities [15]. The gene set “C2.CP.kegg. V6.2.-symbols” [15] was downloaded from the Molecular Signatures Database for GSEA. An adjusted *P* value of < 0.05 was considered statistically significant.

### Constructing a protein–protein interaction (PPI) network and screening hub genes

We used the Search Tool for Retrieving Interacting Genes (STRING) database [16], which predicts PPIs, to construct PPI networks for the selected genes. Genes with scores > 0.4 were selected to construct a network model, which was visualized with Cytoscape V3.7.2 [17]. In the co-expression network, the maximum clique centrality (MCC) algorithm most effectively located the node in a set. The MCC of each node was calculated using CytoHubba plugins [18] in Cytoscape, and genes with the highest eight MCC values were selected as hub genes.

### Constructing a competing endogenous RNA (ceRNA) network based on miRNA-mRNA and miRNA-lncRNA interactions

LncRNA-miRNA interaction data were downloaded from the miRcode database and miRNA-mRNA interaction data were downloaded from the miRTarBase, miRDB, and TargetScan databases. The DESeq2 package of R [12] was used to analyze miRNA and lncRNA expression differences between the high-IRF and low-IRF risk groups. LogFC > 1.0 and *P* < 0.05 were set as criteria for a statistically significant difference. Cytoscape (V3.7.2) was used to construct a ceRNA network by analyzing the correlations between lncRNA- and mRNA-regulated miRNAs simultaneously.

### Tumor immune estimation resource (TIMER) database analysis and comparison of immune-correlation scores between both groups

The TIMER database (https://cistrome.shinyapps.io/timer/) enables users to estimate B-cell, CD4^+^ T cell, CD8^+^ T cell, macrophage, neutrophil, and dendritic-cell infiltration into different tumor types [19]. We used the TIMER database to analyze correlations between the expression levels of different IRF genes and immune cell infiltration in COAD/READ samples.

The R estimate package [20] quantifies immune cell infiltration levels in tumor samples, based on gene expression profiles, and was used to assess the immune activity and stromal score of each tumor sample. Immune cell infiltration levels between both groups were compared using the Mann–Whitney *U* test.

### Analysis of anticancer therapy sensitivity

The Genomics of Drug Sensitivity in Cancer (GDSC) database (https://www.cancerrxgene.org/) enables exploration of gene mutations and targeted cancer therapies. We downloaded gene expression data from cell lines and IC_50_ values to analyze correlations between differentially expressed IRF genes and anticancer drug sensitivities.

### Calculating tumor-mutation load fractions and analyzing genetic variations of IRF family members in CRC

The tumor mutational burden (TMB) of each tumor sample was defined as the number of somatic cell mutations identified, excluding silent mutations. Patients with CRC were divided into high-TMB and low-TMB groups according to the median TMB value. The Wilcoxon rank-sum test was used to compare the risk scores of IRF family genes between both groups.

### Patients and specimens in the validation cohort

Tumor specimens were obtained from 114 CRC patients who underwent treatment at Zhongshan Hospital (Fudan University) between 2008 and 2016. The inclusion criteria were as follows: (a) a clear pathological diagnosis of CRC, (b) complete follow-up data until December 2019, (c) suitable formalin-fixed and paraffin-embedded tissues, and (d) agreeing to participate in the study and provide signed informed consent. CRC diagnosis was based on the World Health Organization criteria, and tumor stages were classified according to the 7th edition of TNM classification of International Union Contra Cancrum. Ethical approval was obtained from the Research Ethics Committee of Zhongshan Hospital. The clinical characteristics of the 102 patients with follow-up data are presented in Additional file 5: Table S1.

### IHC staining evaluation

Cancer and adjacent normal tissues were formalin-fixed, paraffin-embedded, and prepared as tissue microarrays (TMAs) after hematoxylin and eosin staining and histopathology-guided location. Five-micron-thick TMA sections were deparaffinized and rehydrated in 0.1 M citrate buffer (pH 6.0), followed by high-temperature antigen retrieval in a microwave for 15 min. The sections were incubated overnight at 4 °C with primary antibodies against IRF3 and IRF7 (Abcam, Cambridge, UK), CD4 (Servicebio Technology, Wuhan, China), CD8 (Servicebio Technology), CD19 (Servicebio Technology), CD68 (Servicebio Technology), MPO (Servicebio Technology) and CD21 (Servicebio Technology). The sections were incubated for 30 min with a secondary antibody at room temperature and immunostained based on avidin biotin complex formation, using 3,3′-diaminobenzidine. Hematoxylin was used as a counterstain.

Antigen–antibody complexes in whole samples were detected using a panoramic slice scanner (3DHISTECH, Hungary) and viewed with CaseViewer 2.2 (3DHISTECH). H-scores were calculated to evaluate gene expression levels using Quant Center 2.1 (3DHISTECH): H-score = Σ (PI × I) = (% of weakly stained cells × 1) + (% of moderately stained cells × 2) + (% of strongly stained cells × 3), where PI is the proportion of the positive area, and I is the staining intensity.

### Statistical analysis

The data were analyzed with R software (V4.0.2). The independent Student t test was used to estimate the statistical significance of normally distributed variables, and the Mann–Whitney *U* test was used to analyze differences in non-normally distributed variables between two groups of continuous variables. The chi-squared test or Fisher exact test was used to analyze statistical significance between two groups of categorical variables. Correlation coefficients between different genes were calculated via Pearson correlation analysis. The survival package of R was used for survival analysis, Kaplan–Meier survival curves were used to determine survival differences, and the log-rank test was used to evaluate significant differences in survival times between two groups. Univariate and multivariate Cox analyses were used to determine independent prognostic factors. The pROC package of R [21] was used to draw receiver operating-characteristic (ROC) curves, and area under the curve (AUC) values were calculated to assess the accuracy of risk scores for prognosis estimations. All statistical *P* values were bilateral, and *P* < 0.05 was considered statistically significant.

## Results

### Differential transcriptome expression of IRF family in CRC and the prognostic value

**Fig. 1:**
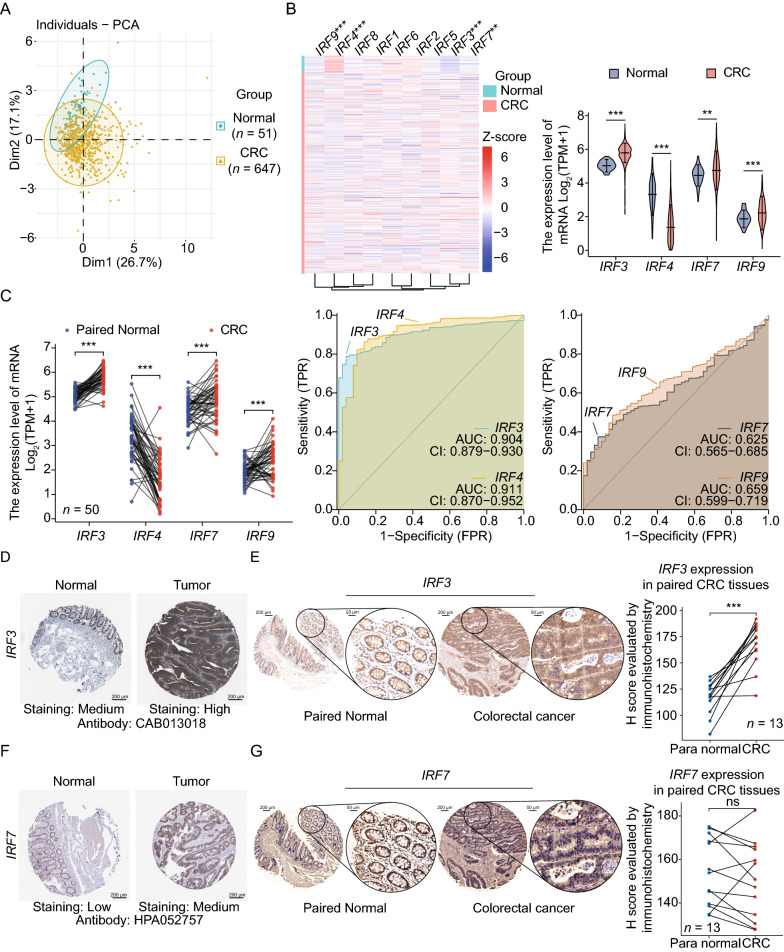
Interferon regulatory factor (IRF) family differentially expressed in patients with colorectal cancer (CRC) from The Cancer Genome Atlas (TCGA) database of COAD/READ datasets. **A** Principal Component Analysis (PCA) based on the expression of the members suggested that the colorectum and CRC could be well separated by the IRF family. **B** A Heat map showed IRF family members differentially expressed in patients with colorectal cancer (CRC). **C** Significant differential expression of IRF3, IRF4, IRF7 and IRF9 were observed between CRC and paired normal cases, with receiver operating-characteristic (ROC) curves suggesting that the expression could help to distinguish tumor and normal tissues. **D, F** Representative immunohistochemistry (IHC) staining from the The Human Protein Atlas database for IRF3 and IRF7 in normal and tumor colorectal tissue were shown. **E, G** IHC analysis of cancer and para-cancerous tissues in 12 patients confirmed the IRF3 and IRF7 protein levels in CRC tissues, revealing that IRF3 was upregulated in CRC, whereas IRF7 did not meet significant statistical level. **p < 0.01; ***p < 0.001

At first, we investigated the transcript level expression of IRF family in pan-cancer of tumor cells as well as tumor tissues in the CCLE and TCGA database (Additional files 3, 4: Figure S3 and S4). Then, principal component analysis of the transcriptomic expression levels suggested that the tumor and normal cases of CRC could be well separated by nine members of IRF family (Fig. 1A). IRF3, IRF4, IRF7 and IRF9 were identified differentially expressing in CRC (Fig. 1B). Specifically, IRF3, IRF7, and IRF9 were significantly upregulated in tumor tissues, whereas IRF4 was downregulated (*P* < 0.001; Fig. 1C). ROC analysis showed that their expression levels had good diagnostic value for CRC (IRF3: AUC: 0.904, IRF4: AUC: 0.911, IRF7: AUC: 0.625, IRF9: AUC: 0.659, Fig. 1C).

Analyzing protein expression levels in CRC and normal tissues with the HPA database (Fig. 1D, F; Additional file 1: Figure S1) revealed that IRF3 and IRF7 were upregulated in cancer tissues. IHC confirmed these results and suggested that the IRF3 protein was more highly expressed in cancer tissues than that in normal tissues (Fig. 1E, G).

### Increased IRF3 and IRF7 expression related to worse prognosis of patients with CRC

**Fig. 2:**
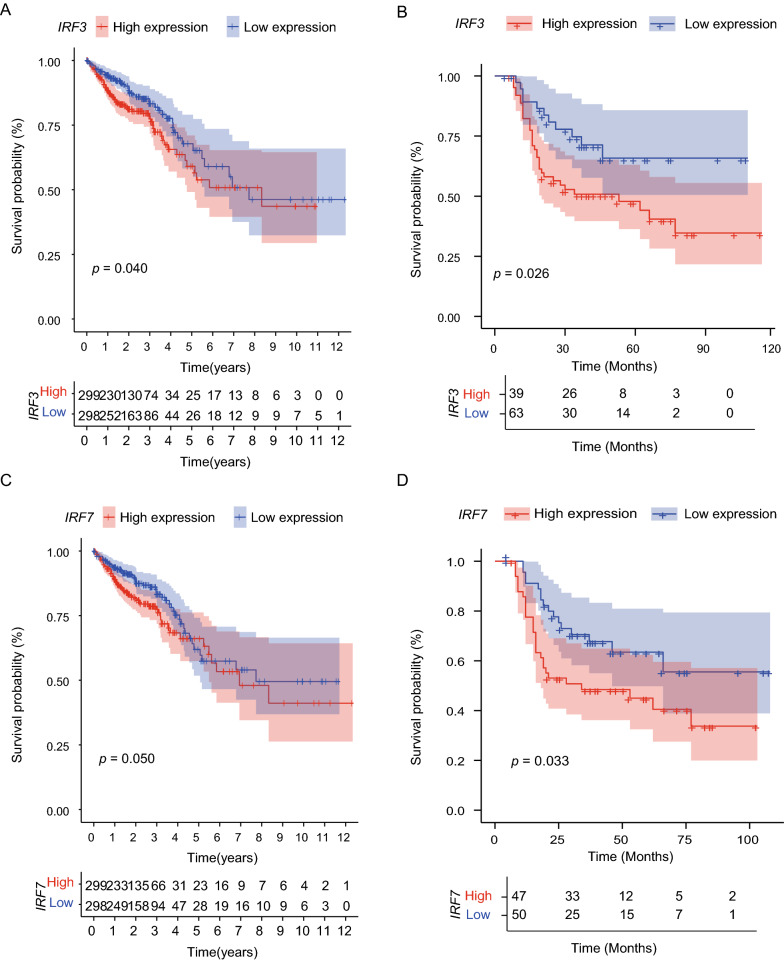
Prognostic value of IRF family in patients with CRC. **A, C** According to TCGA-COAD/READ datasets, patients with high expression level of IRF3 and IRF7 experienced a shorter overall survival (OS). **B, D** High expression level of IRF3 and IRF7 was related to unfavorable OS of 102 cases of CRC by IHC validating

The mRNA expression levels of IRF3 and IRF7 were significantly correlated with OS (Log-rank test, *P* = 0.04 and *P* = 0.05), respectively (Fig. 2A, C). IHC verified these results at the protein level in a cohort of 102 patients with recurrent or advanced CRC. IRF3 and IRF7 protein upregulation showed significant negative correlations with OS (*P* = 0.026 and 0.033), respectively (Fig. 2B, D).

### An IRF risk model predicted OS and DFS in patients with CRC

**Fig. 3:**
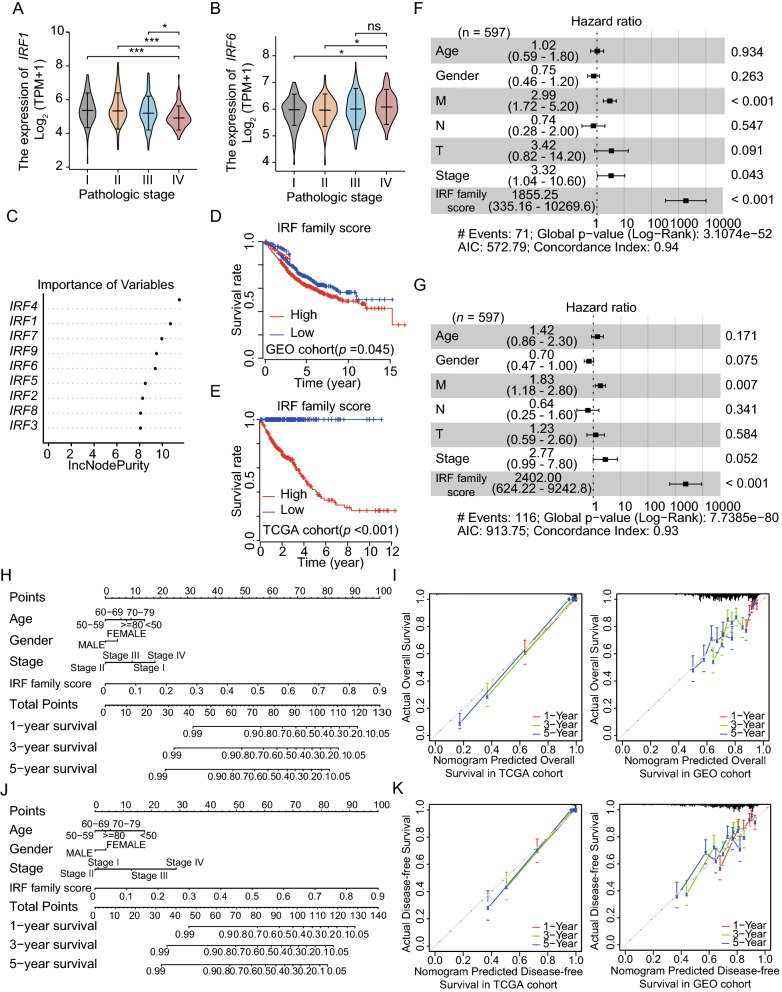
Clinical-prediction models were established based on IRF family expression levels. **A, B** Clinical-correlation analysis showed that IRF1 and IRF6 expression is significantly correlated with different clinical stages. **C** A random-forest model was constructed based on expression levels of IRF family. The weighted values of each member were shown. **D, E** Survival analysis showed that patients with low IRF score had a good prognosis according to data from both The Cancer Genome Atlas (TCGA) and Gene Expression Omnibus (GEO) datasets. **F** The predictive power of multivariate Cox analysis of IRF scores combined with clinicopathological features for OS. **G** The predictive power of multivariate Cox analysis of IRF scores combined with clinicopathological features for predicting disease-free survival (DFS). **H** A histogram for predicting OS based on IRF scores and clinicopathological features. **I** Calibration curve for the OS nomogram; **J** A histogram for predicting DFS based on IRF scores and clinicopathological features. **K** Calibration curve for the DFS nomogram. *p < 0.05; **p < 0.01; ***p < 0.001

We compared IRF expression levels with tumor stages in patients with CRC. IRF1 and IRF6 expression significantly varied (IRF1: *P* < 0.001, Fig. 3A; IRF6: *P* = 0.041, Fig. 3B), whereas the other members of IRF family did not. It was also found that the more advance the clinical stage, the higher the expression of IRF6 (Fig. 3B). Conversely, IRF1 had the highest median expression in stage II patients (Fig. 3A). A random forest model was applied and patients from TCGA and GEO datasets were divided into high- and low-IRF score groups, based on the median risk score (Fig. 3C). Patients in the low-IRF score group showed a better prognosis (TCGA: log-rank *P* < 0.001, Fig. 3D; GEO: log-rank *P* = 0.045, Fig. 3E).

Univariate and multivariate Cox analyses showed that IRF risk score was an independent risk factor for OS and DFS (Tables 2 and 3; Fig. 3F, G). IRF risk scores and clinicopathological features were used to construct a nomogram to predict OS and DFS (Fig. 3H, J). Based on the C-index, the nomogram showed high discriminability in TCGA and GEO datasets (OS: TCGA: 0.928 [0.910–0.945]; GEO: 0.610 [0.571–0.649]; DFS: TCGA: 0.940 [0.922–0.958]; GEO: 0.656 [0.616–0.65]). A calibration curve showed good consistency between the nomograms and the recorded 1-, 3-, and 5-year OS and DFS rates (Fig. 3I, K).

**Table 2:** Univariate and multivariate Cox analyses of overall survival prediction, based on IRF scores calculated by cases from TCGA

Variable	Univariate Cox analysis		Multivariate Cox analysis	
	HR (95% CI)	*P* value	HR (95% CI)	*P* value
Age (≥ 60 vs. < 60)	1.73 (1.09–2.77)	0.020	1.42 (0.86–2.33)	0.171
Gender (male vs. female)	1.08 (0.74–1.56)	0.678	0.69 (0.47–1.04)	0.075
T stage (T3 and T4 vs. T1 and T2)	3.08 (1.50–6.33)	0.002	1.23 (0.59–2.57)	0.584
N stage (N1 and N2 vs. N0)	2.82 (1.93–4.14)	< 0.001	0.64 (0.25–1.62)	0.341
M stage (M1 and MX vs. M0)	2.86 (1.98–4.15)	< 0.001	1.83 (1.18–2.82)	0.006
Stage (III + IV vs. I + II)	3.22 (2.18–4.77)	< 0.001	2.77 (0.99–7.76)	0.052
IRF score (high vs. low)	2216.68 (654.36–7509.12)	< 0.00	2401.99 (624.22–9242.82)	< 0.001

*HR* hazard ratio,* CI *confidence interval

**Table 3:** Univariate and multivariate Cox analyses of disease-free survival prediction, based on IRF scores calculated by cases from TCGA

Variable	Univariate Cox analysis		Multivariate Cox analysis	
	HR (95% CI)	*P* value	HR (95% CI)	*P* value
Age (≥ 60 vs. < 60)	1.00 (0.60–1.69)	0.975	1.02 (0.59–1.78)	0.934
Gender (male vs. female)	1.15 (0.72–1.84)	0.567	0.75 (0.46–1.24)	0.263
T stage (T3 and T4 vs. T1 and T2)	8.40 (2.06–34.31)	0.003	3.41 (0.82–14.17)	0.091
N stage (N1 and N2 vs. N0)	4.77 (2.77–8.24)	< 0.001	0.74 (0.28–1.96)	0.547
M stage (M1 and MX vs. M0)	5.57 (3.45–8.99)	< 0.001	2.99 (1.72–5.18)	0.001
Stage (III + IV vs. I + II)	6.52 (3.57–11.91)	< 0.001	3.32 (1.04–10.61)	0.043
IRF score (high vs. low)	1585.65 (372.03–6758.19)	< 0.001	1855.25 (335.16–10,269.55)	< 0.001

*HR* hazard ratio, *CI* confidence interval

### Relationship between IRF scores and gene expression profiles

**Fig. 4:**
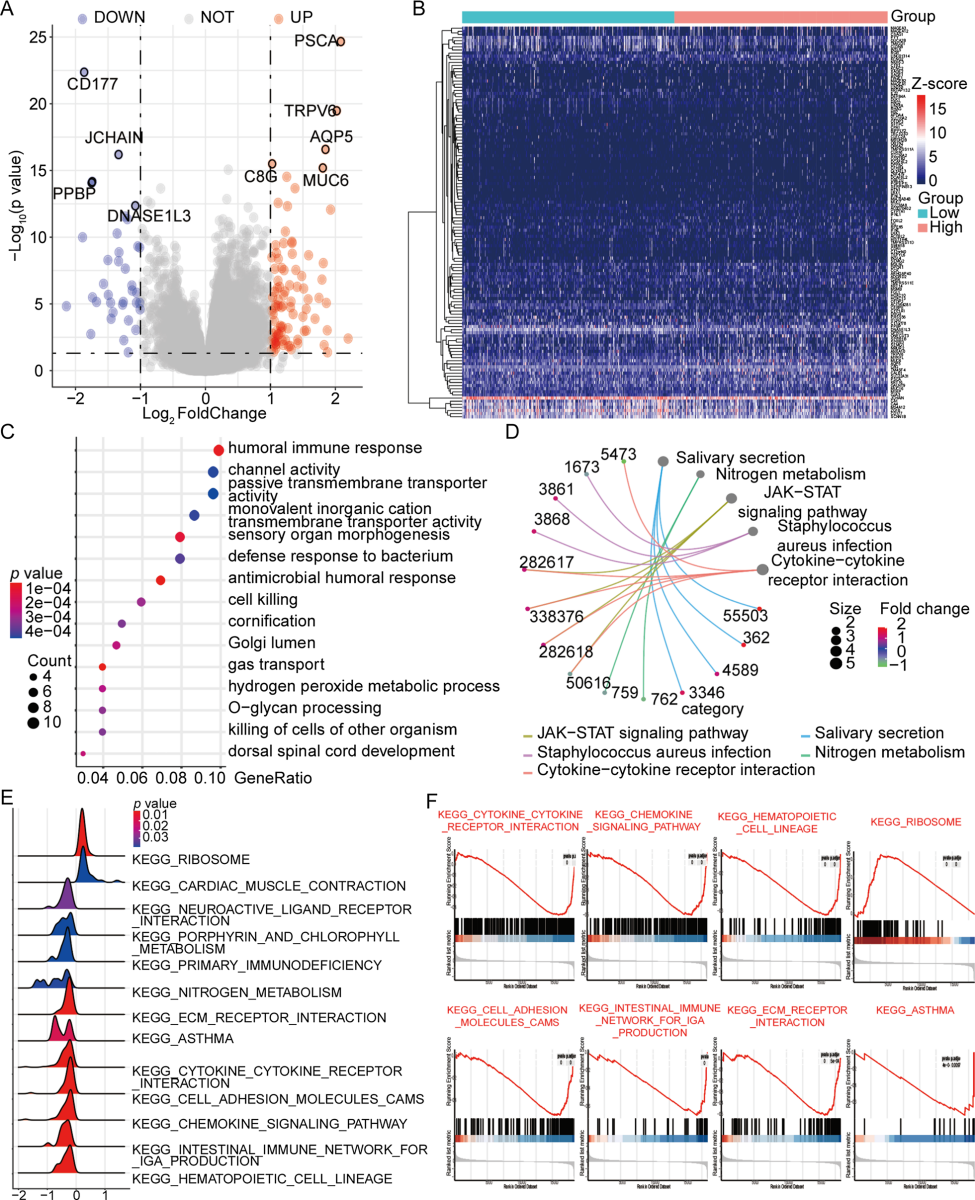
Differentially expressed gene (DEG) and functional-enrichment analysis between high- and low-IRF groups. **A, B** Volcano and heat maps showed 126 DEGs between high- and low-IRF groups. **C** The Gene Ontology analysis suggested that DEGs were closely correlated to the terms such as gas transport, antimicrobial humoral response, humoral immune response, and sensory organ morphogenesis. **D** The five most significant pathways, including Cytokine-cytokine receptor interaction, JAK-STAT signaling pathway, Nitrogen metabolism, Salivary secretion, and *Staphylococcus aureus* infection, and their corresponding gene information were shown after Kyoto Encyclopedia of Genes and Genomes enrichment analysis. **E** Volcanic maps exhibited Gene-Set Enrichment Analysis results for upregulated and downregulated pathways. The X axis represented the relative enrichment score corresponding to the pathway after the Gene Set Enrichment Analysis, and the Y axis represented the names of the most significant pathways obtained by enrichment analysis. **F** Patients in the high-IRF group showed correlations with the terms as ribosome and cardiac muscle contraction pathways, whereas the terms hematopoietic cell lineage, intestinal immune network for IgA production, while chemokine signaling pathway (among other pathways) were significantly underrepresented for patients in the high-IRF group

Analysis of data of patients in the high- and low-IRF score groups identified 126 DEGs (|logFC|> 1.0 and *P* < 0.05; Fig. 4A, B, Additional file 5: Table S2). GO analysis showed that the DEGs were closely related to BP terms such as Gas Transport, Antimicrobial Response, Humoral Immune Response, and Sensory Organ Morphogenesis (Fig. 4C; Additional file 5: Table S3). Differentially expressed IRF genes were associated with enriched KEGG terms such as Nitrogen Metabolism, JAK-STAT Signaling Pathway, *Staphylococcus Aureus* Infection, and Cytokine Receptor Interaction Pathways (Fig. 4D; Additional file 5: Table S4).

GSEA suggested that upregulation of KEGG_RIBOSOME and KEGG_CARDIAC_ MUSCLE_CONTRACTION were significantly enriched, while downregulation of KEGG_ HEMATOPOIETIC_CELL_LINEAGE, KEGG_INTESTINAL_IMMUNE_NETWORK_ FOR_IGA_ PRODUCTION and KEGG_CHEMOKINE_SIGNALING_PATHWAY were significantly enriched in high IRF scores group (Fig. 4E; Additional file 5: Table S5). Figure 4F displays enrichments for the related pathways.

### Expression of IRF family related to tumor immune infiltration

**Fig. 5:**
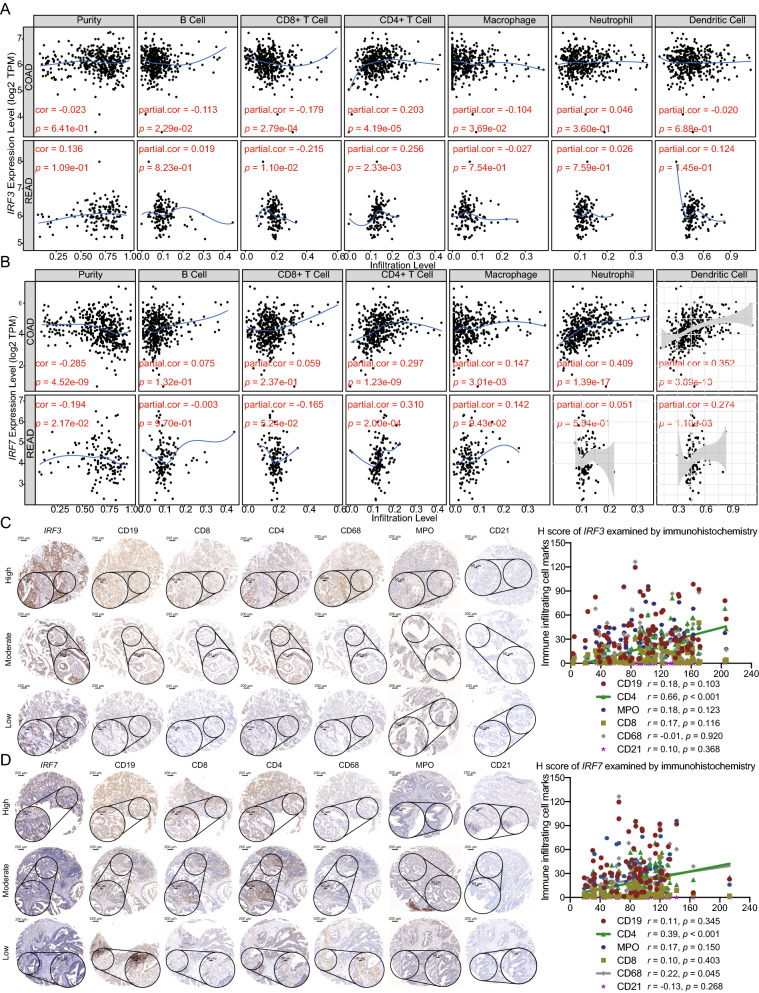
Effects of differentially expressed IRFs on tumor immune infiltration. **A, B** Based on the database tumor immune estimation resource (TIMER), the expression level of IRF3 and IRF7 were associated with tumor immune infiltration in patients with CRC. **C, D** The expression level between IRF3- and IRF7-protein levels and tumor-infiltrating immune cell were evaluated in 102 CRC patients by IHC and the relationships were also investigated

For patients with CRC, IRF expression levels correlated positively, in most cases, with the infiltration levels of different immune cells. The expression of IRF3 in patients with colon cancer was negatively correlated with the infiltration level of B cells, CD8^+^ T cells, and macrophages, and positively correlated with the infiltration of CD4^+^ T cells. IRF3 expression in patients with rectal cancer was negatively correlated with the infiltration level of CD8^+^ T cells, and positively correlated with the infiltration level of CD4^+^ T cells. IRF7 expression was positively correlated with the infiltration of CD4^+^ T cells, macrophages, neutrophils, and dendritic cells in patients with colon cancer; its expression in patients with rectal cancer was negatively correlated with the infiltration level of CD8^+^ T cells, and positively correlated with the infiltration of CD4^+^ T cells and dendritic cells (Fig. 5A, B; Additional file 2: Figure S2). We also observed positive correlations between IRF3 and IRF7 protein expression levels and markers of tumor-infiltrating immune cell via IHC in 102 cases of CRC. IRF3 expression was positively correlated with CD4 expression, suggesting a correlation with CD4^+^ T cell infiltrating, whereas IRF7 expression was positively correlated with CD4 and CD68 expression, suggesting correlations with CD4^+^ T cell and macrophages infiltrating (Fig. 5C, D).

### Correlations between IRF gene expression levels and the biological characteristics of patients with CRC

**Fig. 6:**
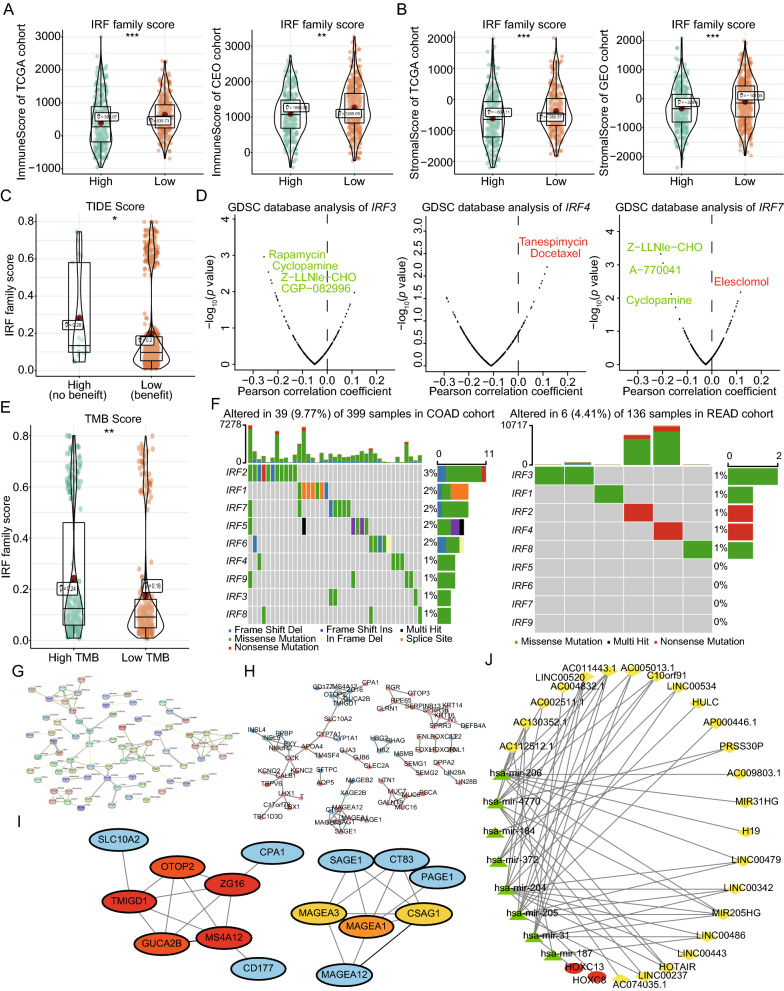
Effects of IRF expression levels on different biological phenomena. **A, B** Based on TCGA and GEO databases, the immune and stromal scores of patients with CRC in the high-IRF group were significantly lower than those in the low-IRF group. **C** Significant differences in IRF risk scores were found between the immunotherapy-benefit and non-benefit groups. **D** The GDSC database was used to evaluate the correlations between IRF family expression levels and sensitivities to chemotherapeutic drugs. The green text indicated negative correlations between IRF expression and sensitivity, and the red text indicated positive correlations. **E** Significant differences in IRF scores between the immunotherapy-benefit and non-benefit groups. **F** Genetic mutation frequencies of IRF family were evaluated in patients from COAD/READ datasets. **G** The Search Tool for Retrieving Interacting Genes (STRING) database was used to analyze a protein–protein interaction (PPI) of DEGs. **H** The STRING results were imported into a Cytoscape software to further depict the contact among them. Red text represented upregulated genes and blue text represented downregulated genes, where the color intensity was positively correlated with fold change. **I** The maximum clique centrality algorithm was used to identify core genes in the PPI network, and the red and yellow nodes represented the top eight hub genes. **J** A ceRNA network was pictured based on differentially expressed mRNAs, miRNAs and lncRNAs, where yellow diamonds represented lncRNAs, green triangles represented miRNAs, and red ovals represented mRNAs. *p < 0.05; **p < 0.01; ***p < 0.001

Analysis of datasets from TCGA and GEO showed that patients in the high-IRF risk group had lower immune and stromal related scores than those in the low-IRF risk group (Fig. 6A, B). Significant differences in IRF scores were found between patients that benefitted from immune therapy and those that did not (Fig. 6C), based on TIDE scores. We analyzed the effects of IRF gene expression levels on sensitivities to different chemotherapeutic drugs or small-molecule inhibitors. In Fig. 6D, red font indicates increased drug sensitivity with increased expression levels of IRF family, and green font indicates negative correlations between drug sensitivity and gene expression levels. Significant differences in IRF scores were also found between patients with high and low TMBs (Fig. 6E). Analysis of TCGA data for mutations in IRF family in patients with CRC showed that the IRF2 exhibited the highest mutation rate (Fig. 6F).

The STRING database was used to construct a PPI network for the DEGs identified in this study (Fig. 6G), which was imported into the Cytoscape software (Fig. 6H). The top eight DEGs were selected from the PPI network as hub genes with CytoHubba plugins, using the MCC algorithm (Fig. 6I). A ceRNA network based on differentially expressed mRNAs, miRNAs, and lncRNAs was established in patients with CRC (Fig. 6J).

## Discussion

Differential expression of IRF genes has been reported in many cancers [6], and IRFs play important roles in CRC tumorigenesis and prognosis. However, this study is the first to explore IRF expression levels at both the mRNA and protein levels, and to determine the prognostic value, effects on immune cells, and potential molecular pathways of IRFs in CRC.

IRF3 and IRF7 are closely related, and unlike other IRFs, they are considered key for evading innate immune responses to virulence factors [22]; thus, they may play crucial roles in anticancer immunity. IRF3 plays important roles in DNA damage responses (DDRs) in cancer [23]. During chemotherapy with DDR agents and immunotherapy involving checkpoint blockade, IRF3 expression is upregulated via cGAS–STING pathway activation [24, 25]. IRF3 activation in response to DDR promotes its role in upregulating RAE1 [26], which is the tumor-cell ligand for NKG2D on NK cells. Together, RAE1 and NKG2D stimulate NK cell-effector function. IRF3 overexpression inhibits tumor-cell growth by increasing p53 activity in vitro [27]. Additionally, IRF3 may be involved in STING activity [28]. Increased PD-L1 expression following treatment with DDR inhibitors is mainly IRF3-dependent [25], and tumor-growth inhibition and immune-checkpoint blockade with DDR inhibitors is completely dependent on the cGAS–STING–IRF3 axis. Our current findings further suggest an additional benefit of cGAS-STING-IRF3 axis activation owing to increased expression of the CXCL10 and CCL5 chemokines, leading to T cell tumor infiltration. Previously, we found that IRF3 and IRF7 could mediate the acquisition of new anti-tumor effector functions in macrophages [29]. In the present study, we observed that high IRF3 and IRF7 expression was related to CD4^+^ T cell, CD8^+^ T cell, B-cell, and macrophage activation, indicating that IRF3 and IRF7 could promote the anticancer effect of immune cells.

Interestingly, among all IRF factors, the mRNA and protein expression levels of IRF3 and IRF7 were significantly upregulated in tumor tissues and associated with poor OS in CRC patients. As IRFs are transcription factors, they may also influence tumor cell development by regulating the transcription of other oncogenes, although the related mechanisms require further investigation. We further assessed the relationship between IRF risk scores and immune and stromal scores in cancer patients to examine why increased IRF3 and IRF7 expression promotes immune cell recruitment without killing tumors. We found that high IRF family score was associated with high TIDE score and high TMB score. It was believed that dysfunction of T cells with high level of infiltration or distinct exclusion of T cells from infiltrating tumors as two primary mechanisms resulting in tumor immune evasion. TIDE is constructed to quantify this effect. Meanwhile, TMB reflects the amounts of mutant proteins brought from neoplasm as well as immunogenic neoantigen load in microenvironment. Hereby, we speculated that IRF family might involve in an imbalance status or even a disorder of immune microenvironment in CRC, more than just attenuating level of tumor immune infiltrations. Since the immune score is calculated by integrating the expression of different immune genes, IRF family involve in the interferon response which represents one type of immune response. Further experimental work is needed to resolve these contradictory results.

Although, we have previously demonstrated that the translation of the IRF2 protein is repressed by microRNA-18 binding to the 3 ′UTR region of the IRF2 mRNA [30], in the present study, we found that the IRF family exhibits a high frequency of genetic variations in the COAD cohort. We therefore constructed a competing ceRNA network containing miRNAs, lncRNAs, and mRNAs expressed at different levels to uncover the underlying regulatory relationships among them. Noncoding RNAs are widely considered to function at every layer of genetic regulation, including duplication, transcription, and translation, especially during cancer development [31]. Fan performed an integrated investigation, constructing a lncRNA-miRNA-mRNA ceRNA network specific to CRC, and identified components related to the prognosis of CRC patients [32]. Qi summarized a comprehensive depiction on the ceRNA crosstalk in CRC [33]. We have also tried to provide novel insights into the connection between coding and noncoding RNAs based on the IRF family, which indicate that HOXC8 and HOXC13, belonging to a highly conserved homeobox family, are regulated by some miRNAs and lncRNAs when mediating the transcription of members of the IRF family. These bioinformatic results point to subsequent experimental validating work.

There are still several limitations in our study. Clinical studies with large sample size are required to verify the predictive value of risk score established by IRF family. The cellular functions and molecular mechanisms of IRF3 and IRF7 in CRC are warrant for conformation with in vitro and in vivo animal experiments. As the biomarker candidates, IRF family should be evaluated in the context of tumor immunotherapy.

## Conclusions

Altogether, we investigated the IRF family in CRC and revealed that the expression of IRF3 and IRF7 were related to tumor immune infiltration as well as prognosis of patients with CRC. The bioinformatic survey provides a basis for future experimental work focusing on these members in CRC.

## Supplementary Information

**Additional file 1: Figure S1.** Representative IHC results of IRF family in normal and CRC tissues were displayed according to the Human Protein Profiling Database

**Additional file 2: Figure S2.** The relationship between members of IRF family and tumor immune infiltrating cells were surveyed in patients from TCGA-COAD/READ dataset.

**Additional file 3: Figure S3.** The mRNA expression levels of IRF family in pan-cancers types of cancer were analyzed using CCLE database.

**Additional file 4: Figure S4.** The mRNA expression levels of IRF family between tumor and paired normal tissues in pan-cancers types were analyzed using TCGA database.

**Additional file 5: Table S1.** The clinicopathological characteristics of 102 patients with CRC.** Table S2**. One hundred and twenty-six differentially expressed genes (DEGs) between low- and high-risk scores according to IRF family.** Table S3**. GO analysis of 126 DEGs based on IRF family scores.** Table S4**. KEGG analysis of 126 DEGs based on IRF family scores.** Table S5**. Results of Gene Set Enrichment Analysis (GSEA).** Table S6**. A comparison of differential risk score group calculated by expression level of IRF family in para normal tissues of patients with CRC from TCGA database.

## Data Availability

All data generated or analyzed during our study are included in the published article or its Additional files.

## Abbreviations

AUC: Area under the curve
BP: Biological process
C-index: Harrell’s consistency index
CCLE: Cell Line Cancer Encyclopedia
ceRNA: Competing endogenous RNA
CI: Confidence interval
COAD/READ: Colon adenocarcinoma/rectum adenocarcinoma
CRC: Colorectal cancer
DBD: DNA-binding domain
DEG: Differentially expressed gene
DFS: Disease-free survival
FPKM: Fragments per kilobase million
GDSC: Genomics of Drug Sensitivity in Cancer
GEO: Gene Expression Omnibus
GO: Gene Ontology
GSEA: Gene set enrichment analysis
HPA: Human Protein Atlas
HR: Hazard ratio
IHC: Immunohistochemistry
IRF: Interferon regulatory factor
KEGG: Kyoto Encyclopedia of Genes and Genomes
logFC: Log fold-change
lncRNA: Long noncoding RNA
MCC: Maximum clique centrality
miRNA: MicroRNA
OS: Overall survival
PPI: Protein–protein interaction
ROC: Receiver operating-characteristic
STRING: Search Tool for Retrieving Interacting Genes
TCGA: The Cancer Genome Atlas
TIMER: Tumor immune estimation resource
TMA: Tissue microarray
TMB: Tumor mutational burden
