# Supplementary figures and images for: Interferon regulatory factor family influences tumor immunity and prognosis of patients with colorectal cancer
Source: J Transl Med. 2021 Sep 6;19:379. doi: 10.1186/s12967-021-03054-3 (PMC8422700; doi:10.1186/s12967-021-03054-3)

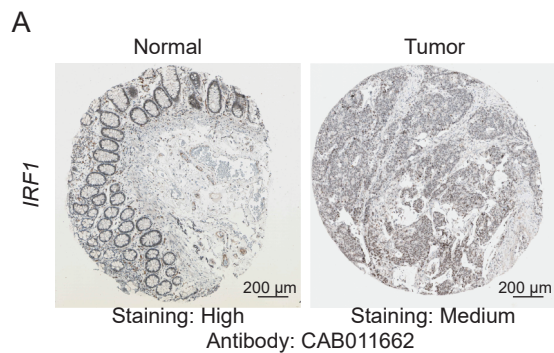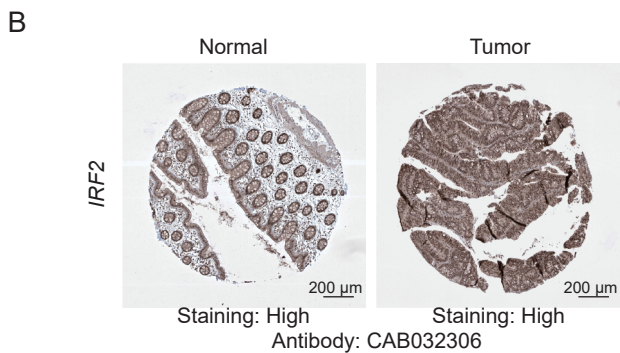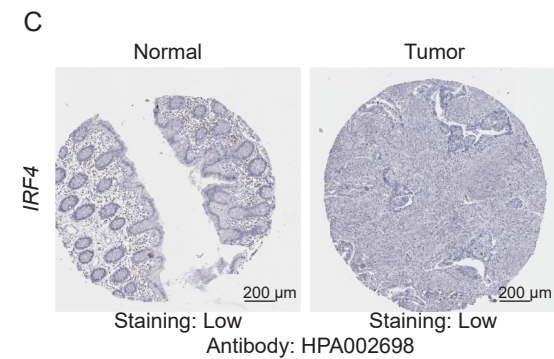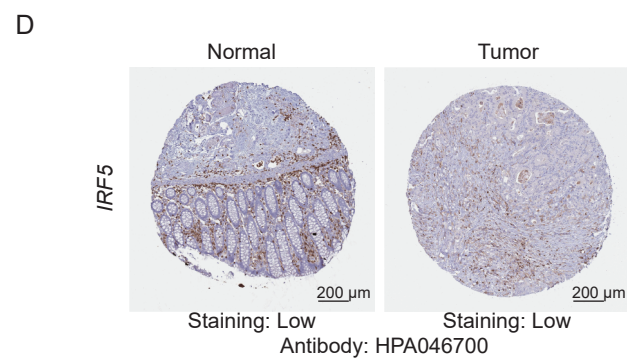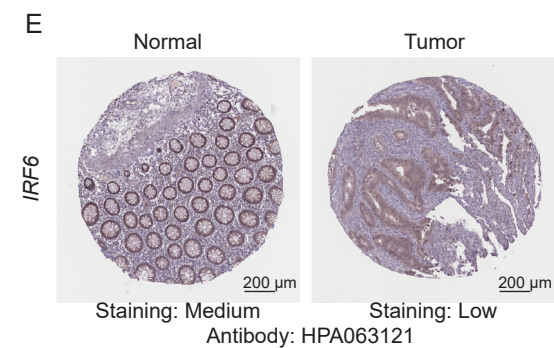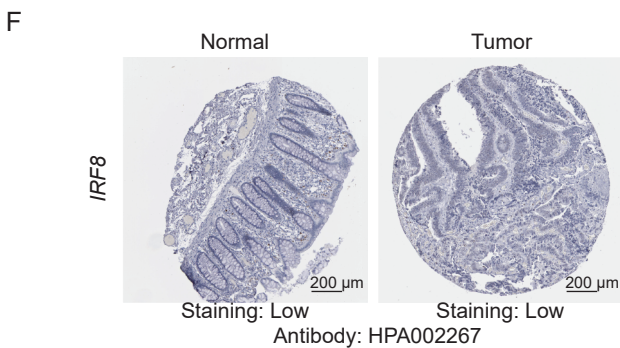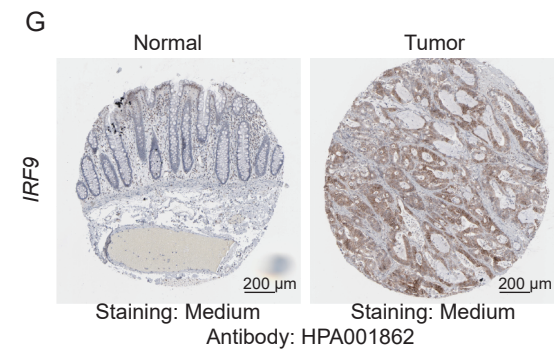

Supplement: Supplementary file 1 — Additional file 1: Figure S1. Representative IHC results of IRF family in normal and CRC tissues were displayed according to the Human Protein Profiling Database [file 12967_2021_3054_MOESM1_ESM.pdf]

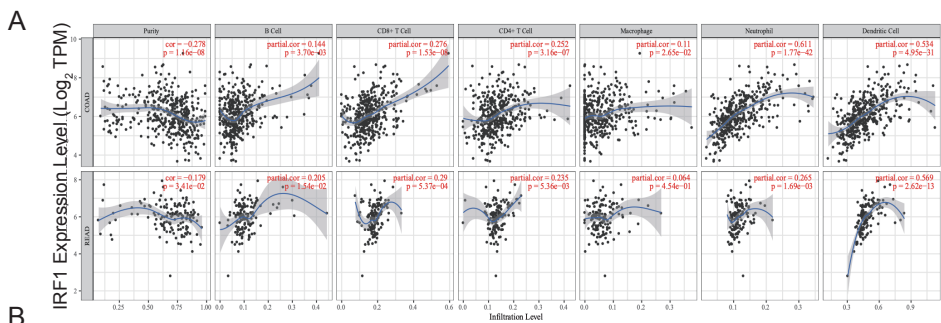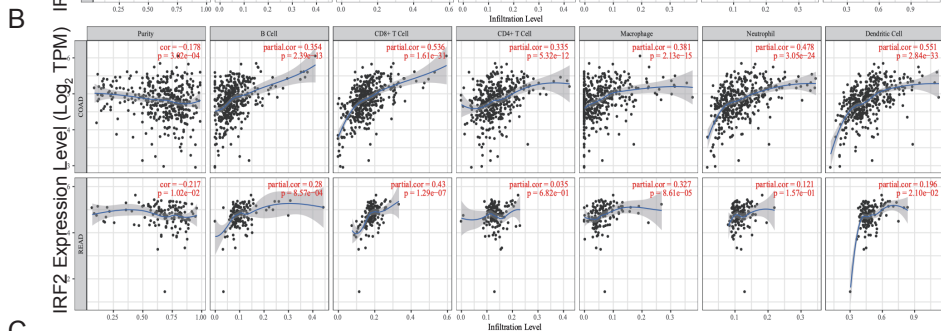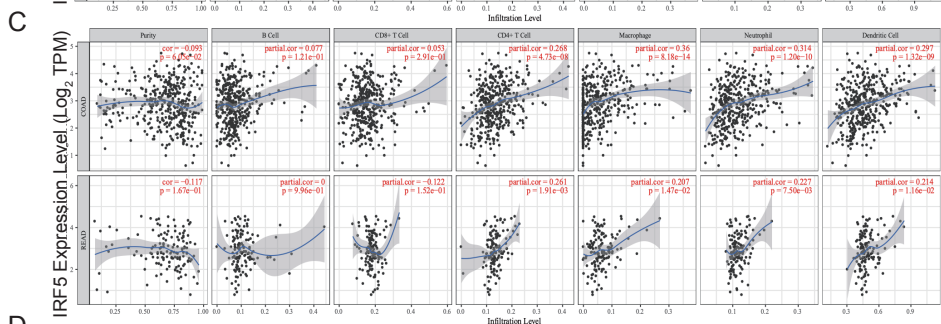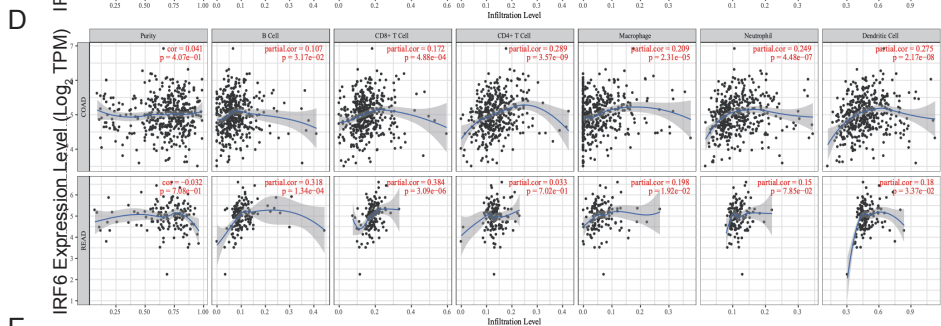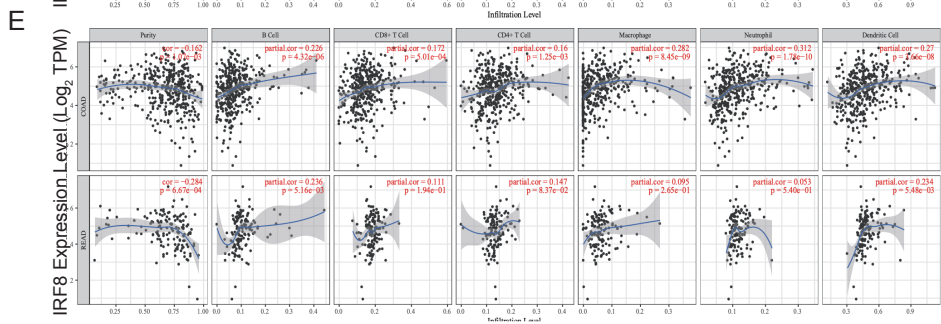

Supplement: Supplementary file 2 — Additional file 2: Figure S2. The relationship between members of IRF family and tumor immune infiltrating cells were surveyed in patients from TCGA-COAD/READ dataset. [file 12967_2021_3054_MOESM2_ESM.pdf]

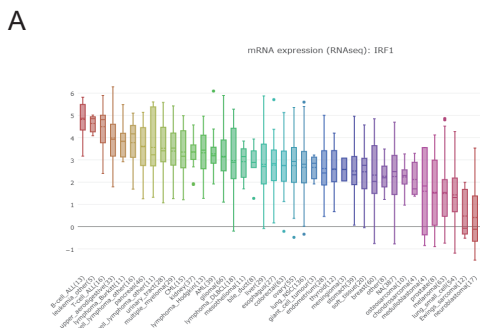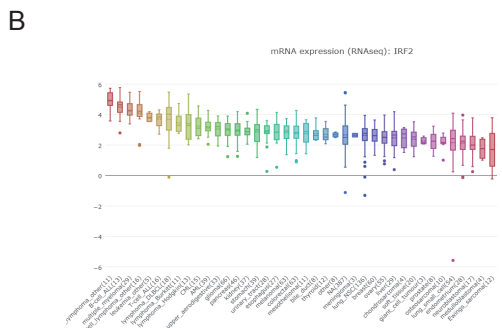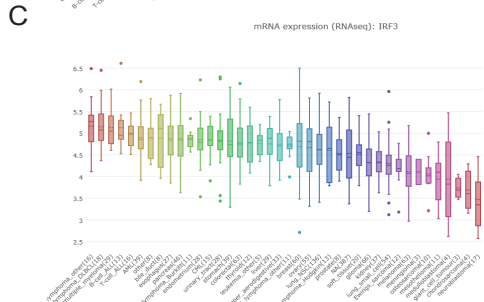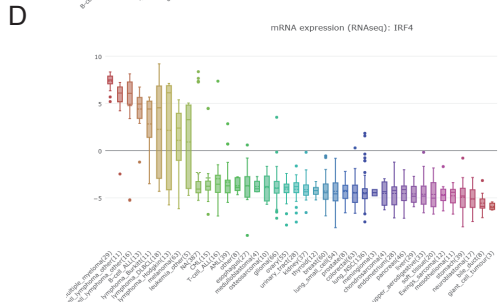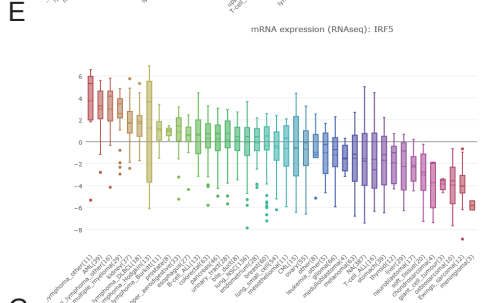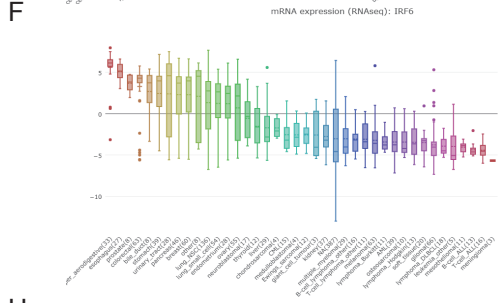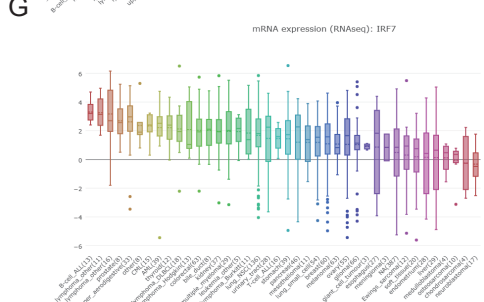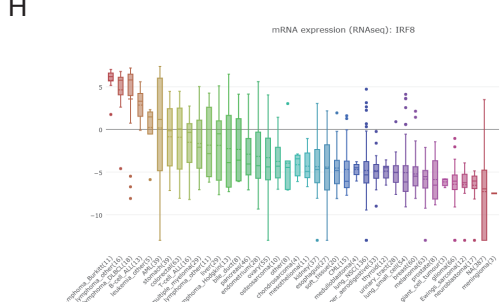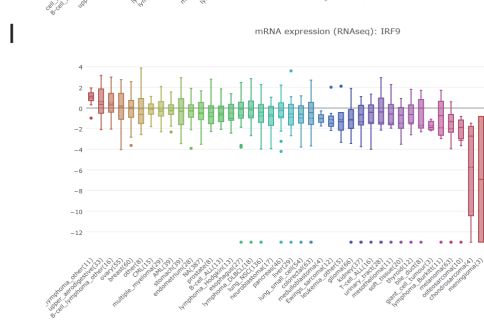

Supplement: Supplementary file 3 — Additional file 3: Figure S3. The mRNA expression levels of IRF family in pan-cancers types of cancer were analyzed using CCLE database. [file 12967_2021_3054_MOESM3_ESM.pdf]

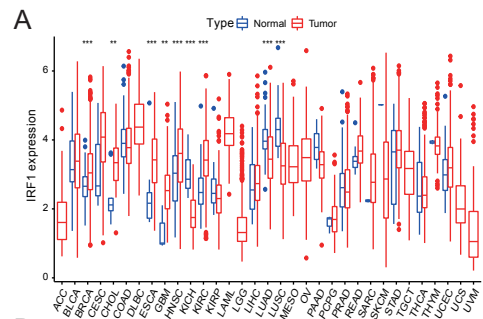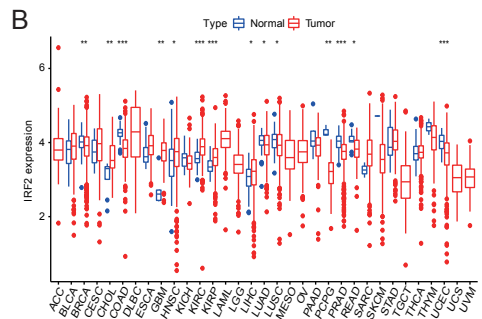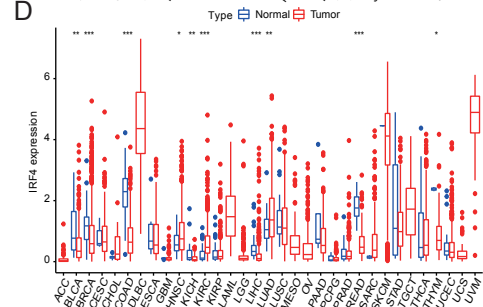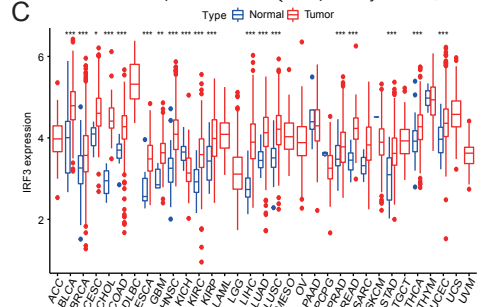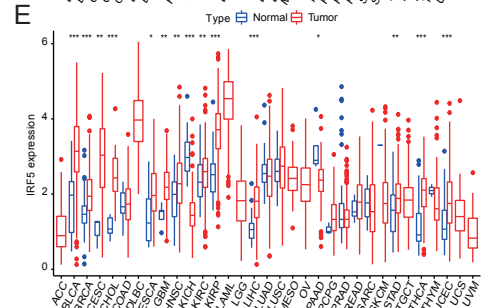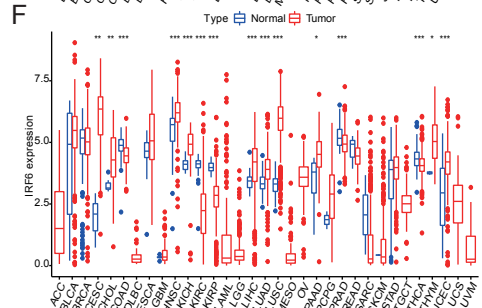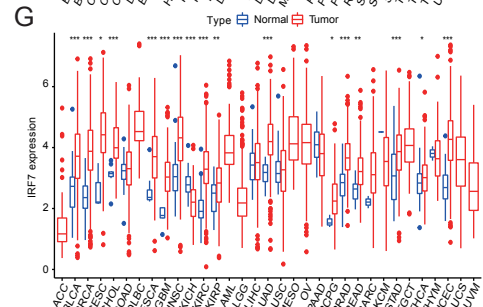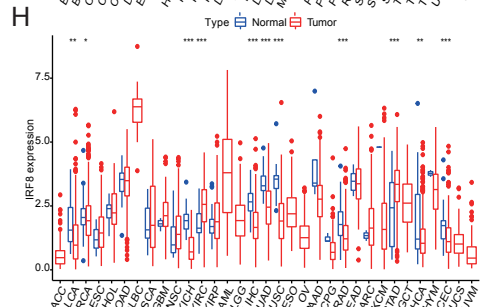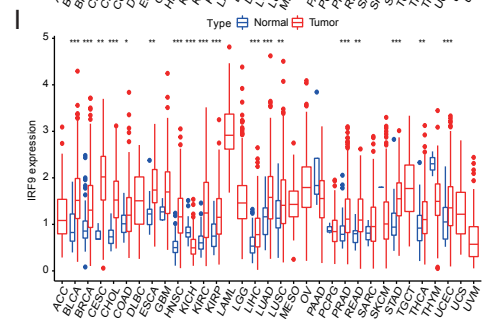

Supplement: Supplementary file 4 — Additional file 4: Figure S4. The mRNA expression levels of IRF family between tumor and paired normal tissues in pan-cancers types were analyzed using TCGA database. [file 12967_2021_3054_MOESM4_ESM.pdf]
