# Supplementary material for: Interferon regulatory factor family influences tumor immunity and prognosis of patients with colorectal cancer
Source: J Transl Med. 2021 Sep 6;19:379. doi: 10.1186/s12967-021-03054-3 (PMC8422700; doi:10.1186/s12967-021-03054-3)
Supplement: Supplementary file 5 — Additional file 5: Table S1. The clinicopathological characteristics of 102 patients with CRC. Table S2. One hundred and twenty-six differentially expressed genes (DEGs) between low- and high-risk scores according to IRF family. Table S3. GO analysis of 126 DEGs based on IRF family scores. Table S4. KEGG analysis of 126 DEGs based on IRF family scores. Table S5. Results of Gene Set Enrichment Analysis (GSEA). Table S6. A comparison of differential risk score group calculated by expression level of IRF family in para normal tissues of patients with CRC from TCGA database. [file 12967_2021_3054_MOESM5_ESM.docx]

**Table S1** The clinic-characteristics of 102 patients with CRC

| **Clinic pathological characteristics** | **Number of Cases/Value** | **%** |
| --- | --- | --- |
| Primary tumor site |  |  |
| Colon | 62 | 60.78 |
| Rectum | 40 | 39.22 |
| Colon location |  |  |
| Right | 30 | 29.41 |
| Left | 72 | 70.59 |
| Gender |  |  |
| Male | 71 | 69.61 |
| Female | 31 | 30.39 |
| Age |  |  |
| less than 60 | 56 | 54.9 |
| more than 60 | 46 | 45.1 |
| Initial stage |  |  |
| Advanced | 53 | 51.96 |
| Recurrence | 49 | 48.04 |
| Survival statues |  |  |
| Death | 48 | 47.06 |
| Alive | 54 | 52.94 |
| Primary tumor resection |  |  |
| Radical | 53 | 51.96 |
| Palliative | 49 | 48.04 |
| Anti-EGFR therapy |  |  |
| Yes | 52 | 50.98 |
| No | 50 | 49.02 |
| Success staining |  |  |
| IRF-3 | 102 | 100.00 |
| IRF-7 | 97 | 95.01 |
| CD19 | 94 | 92.16 |
| CD4 | 96 | 94.12 |
| CD8 | 98 | 96.08 |
| CD68 | 101 | 99.02 |
| MPO | 92 | 90.20 |
| CD21 | 96 | 94.12 |
| Average H score |  |  |
| IRF-3 | 99.22 | / |
| IRF-7 | 91.36 | / |
| CD19 | 37.95 | / |
| CD4 | 20.30 | / |
| CD8 | 8.98 | / |
| CD68 | 23.54 | / |
| MPO | 34.88 |  |
| CD21 | 11.79 |  |
| Median H score |  |  |
| IRF-3 | 101.97 | / |
| IRF-7 | 93.86 | / |
| CD19 | 29.93 | / |
| CD4 | 14.43 | / |
| CD8 | 3.98 | / |
| CD68 | 16.41 | / |
| MPO | 29.91 |  |
| CD21 | 11.12 |  |
| Total | 102 | 100 |

**Table S2** One hundred and twenty-six differentially expressed genes (DEGs) between low- and high-risk scores according to IRF family

| **Gene** | **Log_2_ [Fold Change]** | ***P* value** | **Regulated** |
| --- | --- | --- | --- |
| PSCA | 2.084 | 2.174E-25 | UP |
| CD177 | -1.867 | 4.294E-23 | DOWN |
| TRPV6 | 2.020 | 3.359E-20 | UP |
| AQP5 | 1.848 | 2.631E-17 | UP |
| JCHAIN | -1.337 | 6.393E-17 | DOWN |
| C8G | 1.027 | 3.187E-16 | UP |
| MUC6 | 1.807 | 6.451E-16 | UP |
| M1AP | 1.251 | 2.985E-15 | UP |
| ZG16 | -1.742 | 6.880E-15 | DOWN |
| PPBP | -1.752 | 8.478E-15 | DOWN |
| SLC22A31 | 1.378 | 2.144E-14 | UP |
| GJA3 | 1.119 | 2.686E-13 | UP |
| DNASE1L3 | -1.080 | 4.428E-13 | DOWN |
| TM4SF4 | 1.262 | 4.722E-13 | UP |
| ONECUT3 | 1.926 | 8.546E-13 | UP |
| AC136428.1 | -1.238 | 2.803E-12 | DOWN |
| GPR15 | -1.202 | 4.385E-12 | DOWN |
| KRT16 | 1.229 | 3.753E-11 | UP |
| CA4 | -1.360 | 5.290E-11 | DOWN |
| PRSS56 | -1.892 | 1.011E-10 | DOWN |
| MSLNL | 1.331 | 2.202E-10 | UP |
| ARHGAP40 | 1.333 | 2.690E-10 | UP |
| LINC01314 | 1.203 | 2.775E-10 | UP |
| MYRFL | 1.080 | 4.069E-10 | UP |
| CEBPE | -1.041 | 4.871E-10 | DOWN |
| SCNN1B | -1.012 | 5.910E-10 | DOWN |
| PYY | -1.331 | 1.360E-09 | DOWN |
| GALNT9 | 1.093 | 1.532E-09 | UP |
| RPE65 | 1.337 | 1.757E-09 | UP |
| TMPRSS11E | 1.587 | 2.253E-09 | UP |
| ADGRD2 | 1.021 | 3.211E-09 | UP |
| AC007040.2 | 1.122 | 4.172E-09 | UP |
| OTOP2 | -1.502 | 5.179E-09 | DOWN |
| HBG2 | -1.337 | 6.349E-09 | DOWN |
| KCNC2 | 1.814 | 8.729E-09 | UP |
| KCNQ2 | 1.175 | 2.031E-08 | UP |
| GUCA2B | -1.233 | 2.268E-08 | DOWN |
| GJB6 | 1.067 | 2.332E-08 | UP |
| FOXL2 | 1.391 | 2.863E-08 | UP |
| HOXC10 | 1.210 | 2.953E-08 | UP |
| PGPEP1L | 1.481 | 8.146E-08 | UP |
| CDR1 | 1.794 | 1.503E-07 | UP |
| MUC16 | 1.116 | 1.836E-07 | UP |
| CLDN8 | -1.569 | 3.159E-07 | DOWN |
| NMUR2 | 1.296 | 4.015E-07 | UP |
| IL22 | -1.133 | 4.195E-07 | DOWN |
| CYP7A1 | 1.412 | 5.247E-07 | UP |
| CALB1 | 1.142 | 5.291E-07 | UP |
| DPCR1 | 1.105 | 6.723E-07 | UP |
| LHX1 | 1.513 | 7.035E-07 | UP |
| CA1 | -1.127 | 1.005E-06 | DOWN |
| ANXA10 | 1.223 | 1.182E-06 | UP |
| IFNL1 | 1.066 | 1.216E-06 | UP |
| MS4A12 | -1.058 | 1.476E-06 | DOWN |
| INSL5 | -1.496 | 2.128E-06 | DOWN |
| KRT14 | 1.204 | 2.233E-06 | UP |
| ACTBL2 | 1.033 | 3.161E-06 | UP |
| INSL4 | -1.739 | 3.235E-06 | DOWN |
| APOA4 | 1.747 | 3.567E-06 | UP |
| C12orf40 | 1.580 | 4.518E-06 | UP |
| SEMG1 | 1.116 | 4.822E-06 | UP |
| SLC36A2 | 1.317 | 6.693E-06 | UP |
| SFTPC | -1.677 | 7.155E-06 | DOWN |
| CYP1A1 | -1.264 | 7.159E-06 | DOWN |
| ITLN2 | -1.063 | 8.114E-06 | DOWN |
| MSMB | -1.460 | 8.154E-06 | DOWN |
| MAGEA12 | 1.861 | 8.481E-06 | UP |
| C17orf78 | -1.011 | 8.511E-06 | DOWN |
| FGL1 | -1.275 | 1.120E-05 | DOWN |
| SLC38A8 | 1.078 | 1.220E-05 | UP |
| CCK | 1.194 | 1.392E-05 | UP |
| IFNE | 1.117 | 1.421E-05 | UP |
| MAGEB2 | -2.140 | 1.486E-05 | DOWN |
| SPRR1B | 1.168 | 1.730E-05 | UP |
| CPA1 | 1.309 | 1.774E-05 | UP |
| RGR | 1.077 | 1.830E-05 | UP |
| HIST1H4L | 1.551 | 2.427E-05 | UP |
| TMIGD1 | -1.006 | 2.563E-05 | DOWN |
| SMIM18 | 1.021 | 3.250E-05 | UP |
| KRTAP13.2 | -1.422 | 4.308E-05 | DOWN |
| TMPRSS11D | 1.349 | 5.790E-05 | UP |
| DRGX | 1.041 | 5.870E-05 | UP |
| TSPYL6 | 1.500 | 9.071E-05 | UP |
| LBX1 | 1.687 | 1.297E-04 | UP |
| DLK1 | -1.453 | 1.309E-04 | DOWN |
| LIN28A | -1.185 | 1.735E-04 | DOWN |
| SPRR3 | 1.059 | 1.882E-04 | UP |
| T | 1.152 | 4.422E-04 | UP |
| HOXC13 | 1.182 | 5.110E-04 | UP |
| NKX6.3 | 1.162 | 5.851E-04 | UP |
| CSAG1 | 1.271 | 6.739E-04 | UP |
| DEFB4A | -1.072 | 8.875E-04 | DOWN |
| SERPINB13 | 1.874 | 9.513E-04 | UP |
| CLEC2A | 1.552 | 9.839E-04 | UP |
| SLC10A2 | 1.522 | 1.078E-03 | UP |
| IFNL3 | 1.094 | 1.085E-03 | UP |
| OTOP3 | 1.168 | 1.231E-03 | UP |
| MIA.RAB4B | 1.031 | 1.739E-03 | UP |
| CT83 | -1.765 | 1.846E-03 | DOWN |
| MUC7 | 1.130 | 1.868E-03 | UP |
| SEMG2 | 1.118 | 1.915E-03 | UP |
| MROH2B | 1.090 | 2.890E-03 | UP |
| IVL | 1.062 | 3.666E-03 | UP |
| OR1N2 | 2.196 | 3.944E-03 | UP |
| HTN1 | 1.420 | 4.891E-03 | UP |
| XAGE2 | 1.232 | 5.012E-03 | UP |
| RHAG | -1.257 | 5.263E-03 | DOWN |
| SAGE1 | 1.049 | 5.485E-03 | UP |
| TMPRSS11A | 1.181 | 5.611E-03 | UP |
| DCAF8L2 | 1.020 | 5.741E-03 | UP |
| OR6A2 | 1.093 | 6.276E-03 | UP |
| RIPPLY2 | 1.064 | 7.584E-03 | UP |
| GLYATL3 | 1.070 | 7.832E-03 | UP |
| COX7B2 | 1.422 | 9.235E-03 | UP |
| MAGEA3 | 1.088 | 1.049E-02 | UP |
| CLRN1 | 1.082 | 1.073E-02 | UP |
| MAGEA1 | 1.198 | 1.180E-02 | UP |
| DCAF4L2 | 1.827 | 1.232E-02 | UP |
| LIN28B | 1.302 | 1.411E-02 | UP |
| TBC1D3D | 1.277 | 1.516E-02 | UP |
| UBE2U | 1.082 | 2.216E-02 | UP |
| DPPA2 | 1.322 | 2.502E-02 | UP |
| PAGE1 | 1.013 | 3.278E-02 | UP |
| CFHR4 | 1.052 | 3.835E-02 | UP |
| ZNF479 | 1.879 | 4.230E-02 | UP |
| HBZ | -1.189 | 4.234E-02 | DOWN |

**Table S3** GO analysis of identified 126 DEGs base on IRF family scores

| **ONTOLOGY** | **ID** | **Description** | **Count** | ***P* adjust** | **Gene** |
| --- | --- | --- | --- | --- | --- |
| BP | GO:0015669 | gas transport | 4 | 0.003 | AQP5/HBG2/RHAG/HBZ |
| BP | GO:0019730 | antimicrobial humoral response | 7 | 0.003 | JCHAIN/PPBP/SEMG1/DEFB4A/MUC7/SEMG2/HTN1 |
| BP | GO:0006959 | humoral immune response | 10 | 0.010 | JCHAIN/C8G/PPBP/SEMG1/IFNE/DEFB4A/MUC7/SEMG2/HTN1/CFHR4 |
| MF | GO:0015077 | monovalent inorganic cation transmembrane transporter activity | 9 | 0.020 | SCNN1B/OTOP2/KCNC2/KCNQ2/SLC36A2/SLC10A2/OTOP3/RHAG/COX7B2 |
| MF | GO:0015267 | channel activity | 10 | 0.020 | TRPV6/AQP5/GJA3/SCNN1B/OTOP2/KCNC2/KCNQ2/NMUR2/OTOP3/RHAG |
| MF | GO:0022803 | passive transmembrane transporter activity | 10 | 0.020 | TRPV6/AQP5/GJA3/SCNN1B/OTOP2/KCNC2/KCNQ2/NMUR2/OTOP3/RHAG |
| CC | GO:0005796 | Golgi lumen | 5 | 0.037 | MUC6/ZG16/MUC16/DEFB4A/MUC7 |

**Table S4** KEGG analysis of 126 DEGs base on IRF family scores

| **ID** | **Description** | **Count** | ***P* value** | **Gene** |
| --- | --- | --- | --- | --- |
| hsa04970 | Salivary secretion | 4 | 0.001097 | TRPV6/AQP5/MUC7/HTN1 |
| hsa00910 | Nitrogen metabolism | 2 | 0.003105 | CA4/CA1 |
| hsa04630 | JAK-STAT signaling pathway | 4 | 0.008134 | IL22/IFNL1/IFNE/IFNL3 |
| hsa05150 | Staphylococcus aureus infection | 3 | 0.011706 | KRT16/KRT14/DEFB4A |
| hsa04060 | Cytokine-cytokine receptor interaction | 5 | 0.014519 | PPBP/IL22/IFNL1/IFNE/IFNL3 |
| hsa04979 | Cholesterol metabolism | 2 | 0.025234 | CYP7A1/APOA4 |
| hsa00140 | Steroid hormone biosynthesis | 2 | 0.036436 | CYP7A1/CYP1A1 |
| hsa00830 | Retinol metabolism | 2 | 0.043215 | RPE65/CYP1A1 |
| hsa04610 | Complement and coagulation cascades | 2 | 0.066027 | C8G/CFHR4 |

**Table S5** Results of Gene Set Enrichment Analysis (GSEA)

| **Name** | **Size** | **Enrichment**  **Score** | **NES** | **FDR** | **Leading edge** |
| --- | --- | --- | --- | --- | --- |
| KEGG_CYTOKINE_CYTOKINE_RECEPTOR_INTERACTION | 262 | -0.4667 | -2.07015 | 1.37E-08 | tags=37%, list=13%, signal=32% |
| KEGG_CHEMOKINE_SIGNALING_PATHWAY | 187 | -0.49651 | -2.15541 | 1.37E-08 | tags=32%, list=13%, signal=28% |
| KEGG_HEMATOPOIETIC_CELL_LINEAGE | 81 | -0.63767 | -2.40007 | 3.68E-08 | tags=53%, list=13%, signal=46% |
| KEGG_RIBOSOME | 87 | 0.659957 | 2.079355 | 2.25E-07 | tags=72%, list=22%, signal=56% |
| KEGG_CELL_ADHESION_MOLECULES_CAMS | 112 | -0.52961 | -2.11776 | 6.82E-06 | tags=49%, list=15%, signal=42% |
| KEGG_INTESTINAL_IMMUNE_NETWORK_FOR_IGA_PRODUCTION | 34 | -0.69524 | -2.2379 | 0.000125 | tags=47%, list=9%, signal=43% |
| KEGG_ECM_RECEPTOR_INTERACTION | 83 | -0.50457 | -1.92497 | 0.00046 | tags=48%, list=13%, signal=42% |
| KEGG_ASTHMA | 16 | -0.75445 | -2.06291 | 0.009713 | tags=44%, list=8%, signal=40% |
| KEGG_NEUROACTIVE_LIGAND_RECEPTOR_INTERACTION | 271 | -0.30755 | -1.37737 | 0.02826 | tags=30%, list=11%, signal=27% |
| KEGG_NITROGEN_METABOLISM | 23 | -0.63119 | -1.88066 | 0.036352 | tags=30%, list=4%, signal=29% |
| KEGG_CARDIAC_MUSCLE_CONTRACTION | 73 | 0.525865 | 1.627652 | 0.036844 | tags=37%, list=19%, signal=30% |
| KEGG_PORPHYRIN_AND_CHLOROPHYLL_METABOLISM | 40 | -0.51365 | -1.75836 | 0.036844 | tags=35%, list=13%, signal=31% |
| KEGG_PRIMARY_IMMUNODEFICIENCY | 35 | -0.55336 | -1.81013 | 0.036844 | tags=31%, list=7%, signal=29% |

**Table S6** A comparison of differential risk score group calculated by expression level of IRF family in para normal tissues of patients with CRC from TCGA database.

| **Patients from COAD/READ** | **All patients**  **(n = 51)** | **Low**  **(n = 25)** | **High**  **(n = 26)** | ***P* value** |
| --- | --- | --- | --- | --- |
| Gender |  |  |  | 0.210 |
| Female | 28 (54.9%) | 11 (44.0%) | 17 (65.4%) |  |
| Male | 23 (45.1%) | 14 (56.0%) | 9 (34.6%) |  |
| Age |  |  |  | 1.000 |
| ＜60 | 12 (23.5%) | 6 (24.0%) | 6 (23.1%) |  |
| ≥60 | 39 (76.5%) | 19 (76.0%) | 20 (76.9%) |  |
